# All the menisco‐ligamentary structures of the medial plane play a significant role in controlling anterior tibial translation and tibial rotation of the knee. Cadaveric study of 29 knees with the Dyneelax® laximeter

**DOI:** 10.1002/jeo2.12038

**Published:** 2024-05-28

**Authors:** Baptiste Guegan, Michel Drouineau, Harold Common, Henri Robert

**Affiliations:** ^1^ CHU Rennes: Centre Hospitalier Universitaire de Rennes Rennes France; ^2^ Centre Hospitalier du Haut Anjou Château‐Gontier‐Sur‐Mayenne France

**Keywords:** anterior cruciate ligament, Dyneelax®, medial meniscus, medial side structures

## Abstract

**Purpose:**

This study aimed to determine the respective roles of the anterior cruciate ligament (ACL) and the different components of the medial plane in the control of anterior tibial translation and internal and external tibial rotation.

**Methods:**

Twenty‐nine fresh lower limbs, disarticulated at the hip, were tested in the anatomy laboratory. The following structures were isolated: the ACL, the anteromedial retinaculum (AMR), the medial collateral ligament (superficial and deep MCL), the posterior medial capsule (PMC) and the posterior horn of the medial meniscus (PHMM). The lower limb was positioned at 30° of flexion on the Dyneelax® laximeter (0.1 mm and 0.1° accuracies) and underwent anterior loads up to 200 N and internal and external tibial rotations sectioned from front to back. and the knee was then retested. The results were presented as relative gains in translation and rotations for each structure. Student's *t* test and Wilcoxon tests were used.

**Results:**

The relative gains in translation for the ACL, AMR, superficial MCL, deep MCL, PMC and PHMM, respectively, were 42.9%, 6.7%, 7.4%, 6%, 7.5% and 11.6%. The relative gains in internal rotation for ACL, AMR, superficial MCL, deep MCL, PMC and PHMM, respectively, were 13%, 6.9%, 4.6%, 3.9%, 13% and 8%. The relative gains in external rotation for ACL, AMR, superficial MCL, deep MCL, PMC and medial meniscus, respectively, were 8.9%, 6%, 9.7%, 13.8%,11.2% and 8.5%. All the relative gains in translation, internal and external rotations were significant at each step of transection (*p* < 0.01).

**Conclusions:**

The ligamentous structures of the medial plane constitute a functional unit in which each component has a specific passive contribution. This study highlights the importance of recognising the extent of the medial ligament tears and performing a medial side anatomic and individual reconstruction and a suture of a ramp lesion, in addition to an ACL surgery.

**Abbreviations**
ACL: anterior cruciate ligament
AMR: anteromedial retinaculum
ATT: anterior tibial translation
AV: absolute value
dMCL: deep medial collateral ligament
ER: external rotation
IR: internal rotation
PHMM: posterior horn of the medial meniscus
PMC: posteromedial capsule
POL: posterior oblique ligament
RV: relative value
sMCL: superficial medial collateral ligament

## INTRODUCTION

The anatomical analyses and roles of the different components of the medial side of the knee have long been known [10]. The medial structures consist of the anteromedial retinaculum (AMR), the superficial and deep medial collateral ligament (sMCL and dMCL), the posterior medial capsule (PMC) and the medial meniscus posterior horn (MMPH) [5]. The PMC includes fibres that cross the joint posteromedially, and the posterior oblique ligament (POL) may be identified amongst them [13]. The functions of each of these structures have been described over time [12, 19, 29]. The superficial MCL is the primary passive restraint to the valgus and an important medial restraint to external tibial rotation. The deep MCL is a restraint to external tibial rotation (ER) and the POL is a main restraint to internal tibial rotation (IR). Recent biomechanical studies have confirmed and clarified these data [8, 12, 17, 22, 33]. However, the data on the individual contributions of all the soft‐tissue structures remain incomplete and variable in previous studies [9, 13, 16]. Medial structures lesions are frequently associated with an anterior cruciate ligament (ACL) rupture, in valgus and rotational injuries [13]. Medial collateral ligament injuries were found in 67% of MRI images performed for anterior cruciate ligament rupture [34]. Unhealed or poorly healed medial collateral ligament tears can lead to residual sagittal and rotational laxity, pain and a risk of re‐rupture of an isolated ACL reconstruction [4, 29]. These lesions can be detected by several clinical tests and a meticulous reading of the MRI images. There is currently no consensus on the optimal treatment of severe medial structure lesions or associations with an ACL tear [4]. A better knowledge of the respective functions of the capsular and ligamentous structures of the medial plane could help in their clinical detection and management. To this end, we performed sequential sections of each ligament and recorded anterior tibial translation (ATT) and rotation (IR and ER) displacements with the laximeter Dyneelax® (Genourob Company).

The objective of this study was to determine the respective passive function of the ACL, the different components of the medial side and the MMPH in controlling ATT, IR and ER. The present study intended to identify the primary and secondary restraints in ATT and internal‐external rotation as previously defined [30]. The clinical usefulness of the study was to demonstrate which are the structures that are more important to address in case of multiligament tear is to be treated surgically. We hypothesised that a sequential cutting of the ACL, the medial ligaments, the anteromedial retinaculum, and the posterior horn of the medial meniscus would demonstrate that each structure provides significant passive restraint of ATT and rotations and that there was a synergy between each structure.

## MATERIALS AND METHODS

Twenty‐nine non‐paired, fresh‐frozen cadaver lower limbs (17 female and 12 male) with a mean age of 81 years (range, 41–92 years) were tested in the Anatomic Laboratory of the Medical School of Rennes. Written consent from the donor for their use for educational and research purposes was obtained for each specimen. The lower limbs were collected by disarticulation at the hip and kept frozen at −20°C before the tests. All specimens were thawed at room temperature (25°C) for 1 day before experimentation. During testing, specimens were kept moist with water. The knees were mobilised 10 times in flexion, extension and rotations to ensure that they were flexible and able to flex to at least 130°. The knees were stable for clinical testing in frontal and sagittal planes. Inclusion criteria were stable and mobile knees, and exclusion criteria were signs of ligamentous injuries, bone abnormalities, severe osteoarthritis, or scars indicating previous surgery. All the knees had an X‐ray (coronal and sagittal planes) before dissection. Six knees out of 35 initially selected were rejected by the surgeon who performed the dissections. After testing, all specimens were checked to ensure the integrity of the cartilage.

### Dissection technique

**Figure 1:**
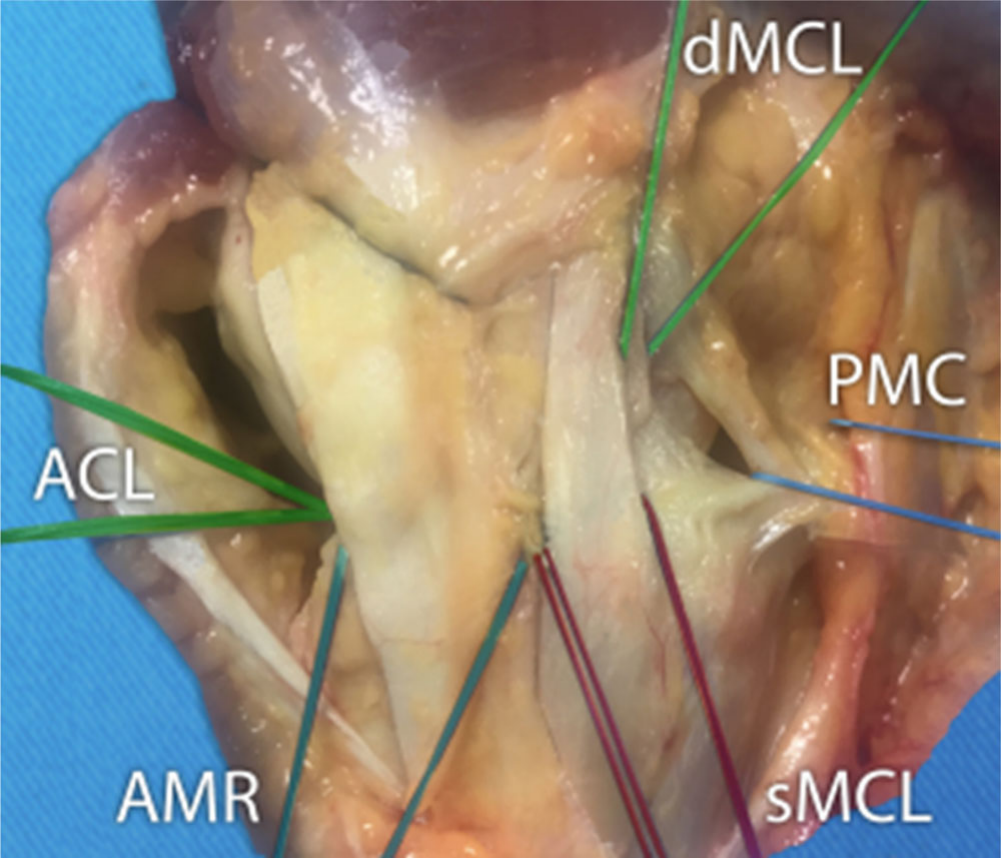
Medial view of a right knee. The identification starts in the middle third as it represents the medial collateral ligament (MCL). This is divided into superficial (sMCL) and deep layers (dMCL). The anterior margin of this structure was clearly defined by palpation of longitudinal fibres but posteriorly the margins were less clear. At the posterior margin of the middle section, the longitudinal fibres blended with oblique fibres coming from the posterior third. This third part is composed of three layers: the facial layer (layer 1), the sMCL (layer 2) and the dMCL and the capsule (layer 3) according to Warren and Marshall's description. The MCL is attached on and around the femoral epicondyle. The anterior third named anteromedial retinaculum (AMR) is composed of fascia (layer 1) and capsule (layer 3) without substantial ligament structure linking the femur to the tibia. In the posterior third, layers 2 and 3 were not separated and formed the posterior medial capsule (PMC). The localised thicker band in the PMC is the posterior oblique ligament (POL). ACL, anterior cruciate ligament.

An orthopaedic surgeon (H.R.), experienced in knee surgery, performed all the dissections. A long medial arciform incision exposed the medial side, taking care to preserve the ligaments and joint capsule intact. The skin and subcutaneous tissues were removed. The medial aspect of the knee from the edge of the patellar tendon to the head of the medial gastrocnemius muscle was divided into three parts (Figure 1) [21]. The identification and separation of the three parts were done above the medial joint line. The identification starts in the middle third as it represents the MCL. This is divided into superficial (sMCL) and deep layers (dMCL). The anterior margin of this structure was clearly defined by palpation of longitudinal fibres but posteriorly the margins were less clear. At the posterior margin of the middle section, the longitudinal fibres blended with oblique fibres coming from the posterior third. This third part is composed of three layers: the facial layer (layer 1), the sMCL (layer 2) and the dMCL and the capsule (layer 3) according to Warren and Marshall's description [31]. The MCL is attached to the femoral medial epicondyle [21]. The anterior third named AMR is composed of fascia (layer 1) and capsule (layer 3) without substantial ligament structure linking the femur to the tibia. In the third posterior, layers 2 and 3 were not separated and formed the PMC. The localised thicker band in the PMC is the POL [13]. The synovial junction to the posterior segment of the medial meniscus was identified over a length of 2 to 3 cm. The whole ACL was dissected at the midportion through an anteromedial vertical arthrotomy. No. 2 Vicryl sutures were tied around each tested structure: ACL, AMR, sMCL, dMCL, PMC and the MMPH. All the muscles and soft tissues were kept intact.

### The laximeter Dyneelax®

**Figure 2:**
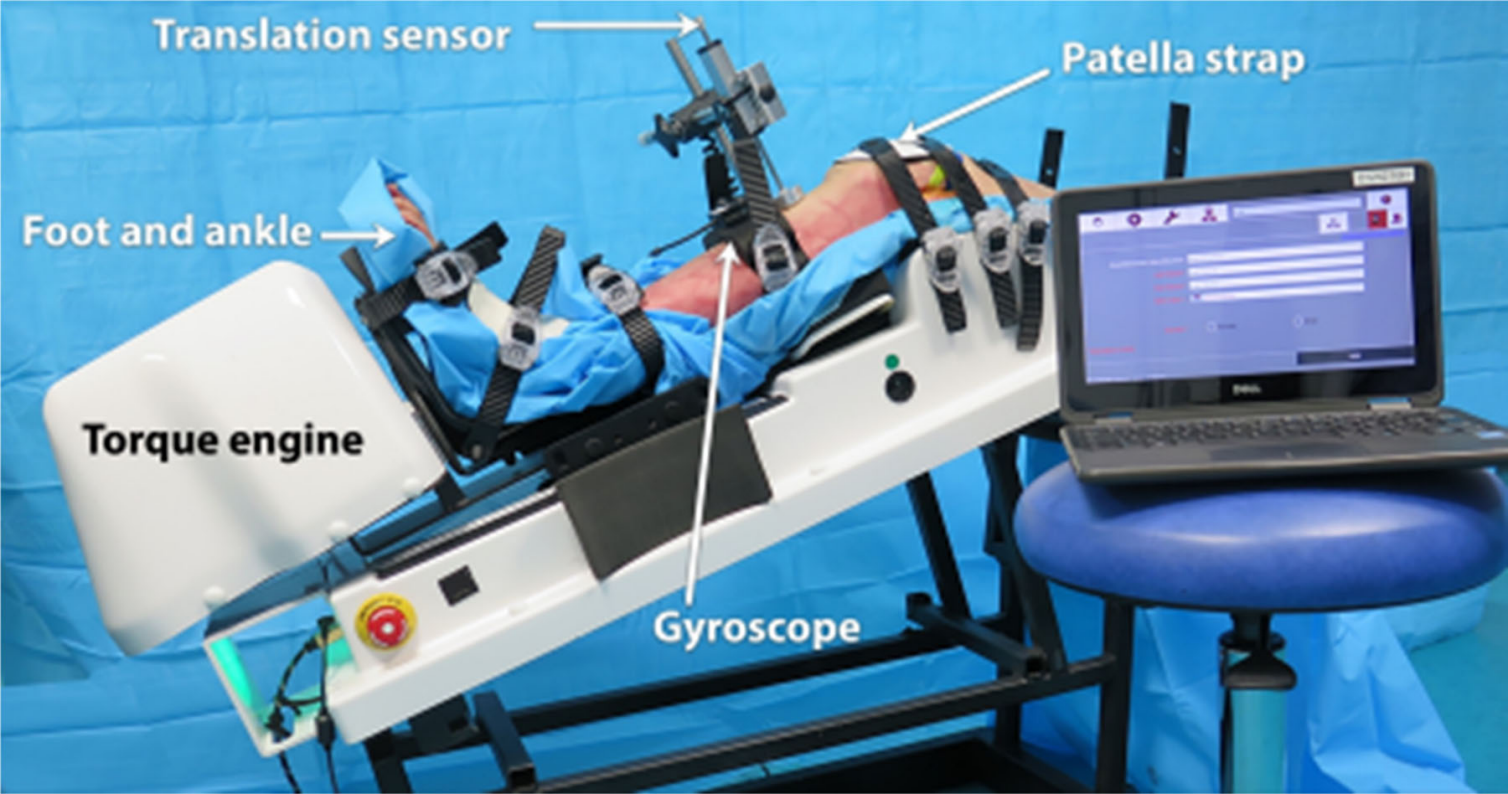
Dyneelax®. The Dyneelax allows anterior tibial translation under loads up to 250 N and tibial rotation (internal and external) up to 8 N m separately. The lower limb was placed on a thermoformed support at 30° of flexion. The femoral head was secured horizontally by a transverse rod. The foot and ankle were attached to a dual bootstrap, providing a stationary block under the tibia. Anterior tibial translation (ATT) is produced by a linear jack and axial torque (internal tibial rotation [IR] and external tibial rotation [ER]) by a rotation engine. ATT were registered by a translation sensor and rotations (ER and IR) by a gyroscope.

We used a non‐invasive and static translational and rotational knee laximeter, the Dyneelax® (Figure 2). The Dyneelax® is derived from the Rotam® and the GNRB®, which have been validated and widely used in biomechanical works and clinical practice for many years [6, 18, 20, 23]. The precision (repeatability) of the Dyneelax® was up to 0.1 mm and up to 0.1° [6]. Dyneelax® allows anterior tibial translation (ATT) under forces up to 200 N and rotatory torque up to 5 N m, separately from the translation. The lower limb was placed on a thermoformed support at 30° of knee flexion. The femoral head was secured horizontally by a transverse rod (diameter 8 mm) to prevent any rotation of the femur. The foot and ankle were attached to a dual bootstrap, providing a stationary block under the tibia. The initial knee position (position zero) was defined by ‘the patella at the zenith’ and the foot‐ankle block was in a natural resting position of the leg (usually in slight external rotation) controlled by the absence of constraint on the boot sensors. The initial position of the lower limb obtained after complete stabilisation was not further modified during all the recording stages. The induced ATT (load up to 200 N) and rotations torque (up to 5 N m) were measured from this neutral position (femur fixed) and were recorded. All specimens were subjected to the same loads (200 N) and secondarily to the torques (5 N m).

### Testing protocol

The intact knee was tested first, followed by sequential complete transections from front to back in the stated order: ACL (28), AMR (28), sMCL (20), dMCL (20), PMC (29) and ultimately the MMPH (16) while the knee remained mounted in the device, at 30° of flexion. The ACL was transected through a 5 cm longitudinal anteromedial arthrotomy (not sutured before the tests) and the medial structures were cut transversely at the level of the superior rim of the medial meniscus with the help of No. 2 Vicryl sutures, doubled up and used in the same manner as a Gigli saw. The MMPH tear at the meniscocapsular junction was made through a longitudinal posteromedial arthrotomy. The vertical section of the medial meniscus (mean length of the tear measured 25 mm) simulated a ramp lesion [27]. To account for any viscoelastic effects of the tissues, all measurements were recorded five times and the mean data were taken as the result in each test. The results were registered as translation curves in millimetres and torque curves (internal and external rotational laxity) in degrees (absolute values) of the tibia relative to the femur. We calculated the increase in % (relative values) for each transected ligament and meniscus relative to the section of all studied structures.

### Statistical analyses

A power calculation (G*Power) based on ER at 30° of flexion determined that a minimum sample size of 26 knees would be needed to detect a significant change in laxity of 1° between the intact and cut state with 80% power and 95% confidence intervals [2, 32]. Statistical analyses were performed using SAS software, version 9.4 (Windows). The results were expressed in absolute values (AV in mm and in degrees) as the difference between the first state (ACL sectioned) and the intact knee, then between the second state (Anterior cruciate ligament and anteromedial retinaculum sectioned) and the first state and so on until sectioning of the ACL, the entire medial plane and the MMPH [19, 21]. The results were also expressed in relative values (RV in %) as the % of increase after the cut of one structure relative to the intact knee before the section. *Q*–*q* plot graphics were used as a test of normality. Comparisons on paired data were carried out using a paired Student's *t* test if the data followed normal distributions, and Wilcoxon Signed Rank Test if the data did not follow normal distributions. Significance was set at *p* < 0.05.

## RESULTS

**Figure 3:**
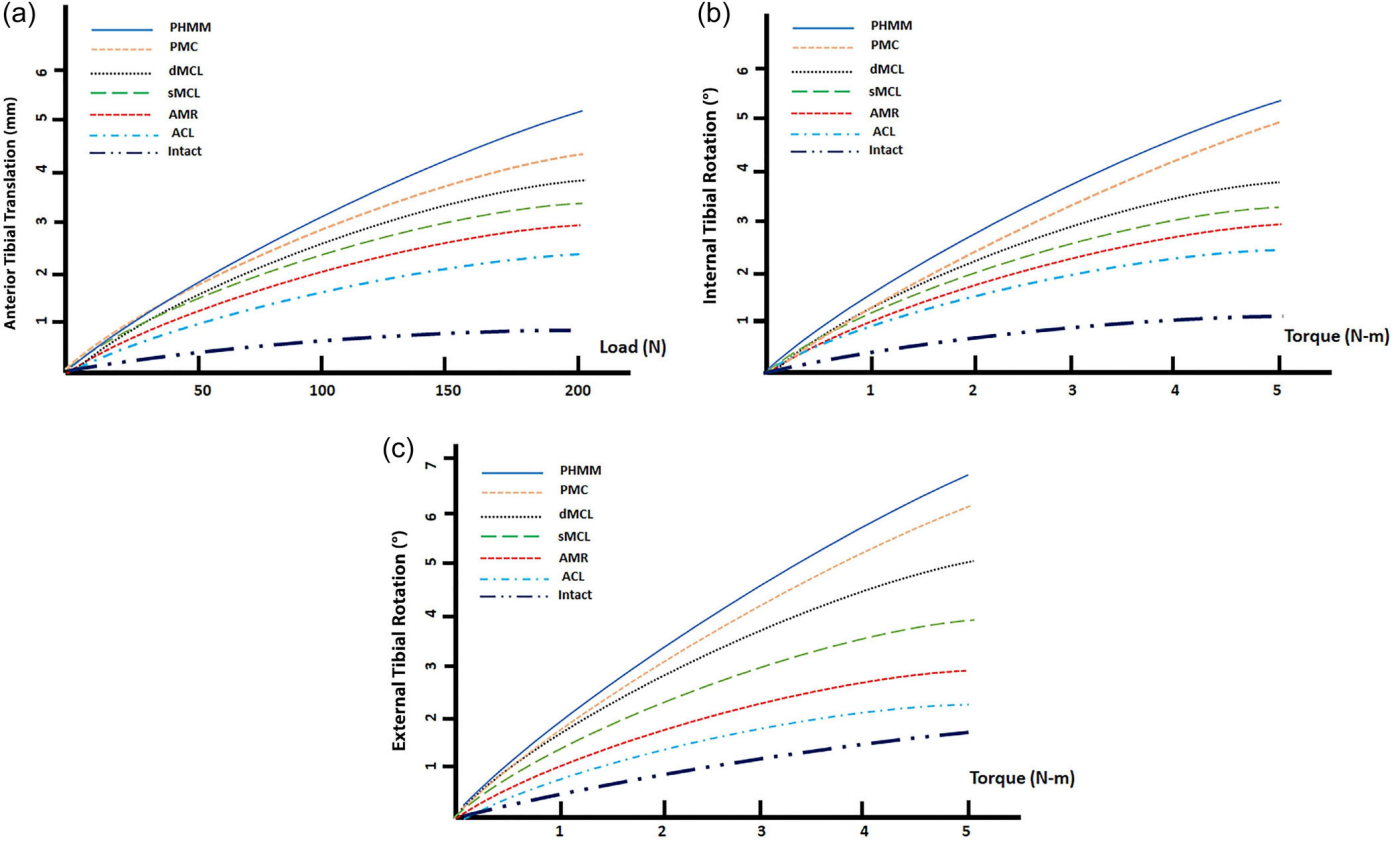
Results in anterior tibial translation (a), internal tibial rotation (b) and external tibial rotation (c) after each transection: anterior cruciate ligament, anteromedial retinaculum, superficial medial collateral ligament, deep medial collateral ligament, posteromedial capsule, posterior horn of the medial meniscus at 30° of flexion.

The gains in AV (ATT, IR and ER) were significant (*p* < 0.01) at each step of transection (Table 1). The gains in RV in ATT (200 N) for the ACL, AMR, sMCL, dMCL, PMC and MMPH, respectively, were 42.9%, 6.7%, 7.4%, 6%, 7.5% and 11.6% (Figure 3a). The relative gains in IR (5 N m) for ACL, AMR, sMCL, dMCL, POMC and MMPH, respectively, were 13%, 6.9%, 4.6%, 3.9%, 13.1% and 8% (Figure 3b). The relative gains in ER (5 N m) for ACL, AMR, sMCL, dMCL, PMC and MMPH, respectively, were 8.9%, 6%, 9.7%, 13.8%, 11.2% and 8.5% (Figure 3c). After sectioning all the medial structures, ATT mean gain in AV was 2.9 mm, IR mean gain was 4° and ER mean gain was 6°. If we combine the ACL section, the translation mean gain was 5.8 mm, the IR mean gain was 5.3° and the ER mean gain was 7° (Table 1).

**Table 1:** Results for the anterior tibial translation (ATT), internal rotation (IR) and external rotation (ER) in absolute (mm) and relative values (%).

	ATT			IR			ER		
	AV (mm)	*p*	RV (%)	AV (°)	*p*	RV (%)	AV (°)	*p*	RV (%)
**ACL**	2.9	*p* < 0.01	42.9	1.4	*p* < 0.01	13	1	*p* < 0.01	8.9
**AMR**	0.5	*p* < 0.01	6.7	0.8	*p* < 0.01	6.9	0.7	*p* < 0.01	6
**sMCL**	0.5	*p* < 0.01	7.4	0.5	*p* < 0.01	4.6	1.4	*p* < 0.01	9.7
**dMCL**	0.5	*p* < 0.01	6	0.5	*p* = 0.01	3.9	1.8	*p* < 0.01	13.8
**PMC**	0.5	*p* < 0.01	7.5	1.5	*p* < 0.01	13	1.3	*p* < 0.01	11.2
**PHMM**	0.9	*p* < 0.01	11.6	0.7	*p* < 0.01	8	0.8	*p* < 0.01	8.5
**Total**	5.8			5.3			7		

Abbreviations: ACL, anterior cruciate ligament; AMR, anteromedial retinaculum; AV, absolute value; dMCL, deep medial collateral ligament; PHMM, posterior horn of the medial meniscus; PMC, posteromedial capsule; RV, relative value; sMCL, superficial medial collateral ligament.

## DISCUSSION

The most important finding of our study was that all structures of the medial side were involved in sagittal and rotational control of the knee. This confirms our hypothesis that each of the structures in the medial plane had its own role in knee translation or rotation control. As previously defined in the literature, ‘primary restraint’ resists most of force in a given direction, while ‘secondary restraint’ contributes to the stability after the primary restraint has been removed [24].

In the present study, the ACL was the primary restraint to anterior translation and the secondary restraint to internal rotation, the secondary restraint for anterior translation was the MMPH. The PMC was the primary restraint for internal rotation. For external rotation, dMCL was the primary restraint and the sMCL was the secondary restraint.

Numerous biomechanical studies confirm the role of the ACL as a primary brake of ATT and IR [2, 7, 23, 24].

The role of the AMR has been little studied from a mechanical point of view, but we found that it plays a significant role, particularly in IR (6.9% gain) and ER (6% gain). Herbst et al. demonstrated an important contribution of AMR in resisting ER (20% in flexion 30°) and ATT in ER (12% in flexion 30°) [3]. AMR ruptures have been consistently found in medial plane sprains operated on acutely or chronically, in association with variable ACL and MCL lesions [25].

According to Griffith et al., sMCL is the primary restraint to valgus at all angles of flexion, to ER at 30° of flexion and to IR at all angles of flexion [9]. Several studies have confirmed the role of sMCL in controlling valgus and IR [21, 30]. Other studies confirmed the primary braking role on the ER at 30° flexion [2, 8, 21, 32]. Our study confirmed the major role of sMCL in ER control (gain of 1.4° or 9.7%), which is slightly less than the gains observed by Robinson et al. (3°) and Warren et al. (2°) [21, 31]. The sMCL had only a secondary role in IR in our study (gain of 0.5° or 4.6%), as in the study by Griffith et al. [9]. The present study found a significant increase in ATT after sectioning the sMCL (gain of 0.5 mm or 7.4%), as did a previous study [2].

The dMCL contributes secondary restraint to valgus stress [2, 8]. The dMCL was the primary restraint to ER at 30° of flexion for Ball et al. [2]. For many authors, the meniscofemoral division acted as an important restraint to ER between 30° and 90° of flexion [8, 33]. In our study, the dMCL acted as a primary brake in RE (mean gain of 1.8° or 13.8%), in line with many publications [30, 33]. The function of the dMCL could be superimposed to the anterolateral ligament at the lateral side of the knee. Today, an extra‐articular tenodesis is largely indicated in addition to an ACL reconstruction to protect the graft during the ligamentisation steps. In case of an anteromedial rotatory instability (AMRI), an equivalent medial procedure could logically be indicated to reduce loads on the ACL graft during the healing phase. This important role of the dMCL in ER confirms the importance of dMCL reconstructions associated with that of the ACL in AMRI. This reconstruction of the dMCL in AMRI could protect the ACL plasty during ligamentisation [2, 26]. The present study demonstrated a major function of the two components of the MCL in the restraint of ER (gains de 3.2°, meaning 23.5%) and a not insignificant function in the control of the ATT (mean gain 1.1 mm or 13.4%). The literature is more controversial regarding the role of dMCL in IR. Griffith et al. then Laprade et al. reported that the meniscofemoral component of the dMCL was a secondary restraint in IR at 0° and 30° flexion [9, 16]. In contrast, Robinson et al. were unable to demonstrate an IR resistance function for the dMCL meniscotibial component [22]. Our study shows a weak role for dMCL in IR control (mean gain of 0.5° or 3.9%).

Multiple studies have recognised the PMC as a primary restraint to IR at all angles of flexion (excluding the ACL) and a secondary restraint to external rotation at 30° of flexion [9, 16, 22, 30, 32]. With the knee close to extension, IR of the tibia caused the oblique fibres of the PMC to tighten further. Wijdicks et al. described a reciprocal function between the PMC and the sMCL [33]. The PMC provides more internal restraint in extension while the sMCL contributes more restraint in higher degrees of flexion [33]. The PMC is also involved in valgus stabilisation, which we have not tested [8, 30]. Studies by Ball et al. and Sullivan et al. found little increase in ATT after sectioning the PMC, but for Griffith et al., sectioning the ACL followed by the PMC increased ATT [2, 9, 28]. Our study found a significant role for the PMC in ATT control (gain of 0.5 mm or 7.5%), confirmed the PMC's major role in IR control (gain of 1.5° or 13%) and a lesser role in ER control (gain of 1.3° or 11.2%), at 30° flexion. Simultaneous involvement of the LCM and PMC (‘Posteromedial corner tear’) increases the anterior drawer and RE in the laboratory. This anteromedial rotatory instability (AMRI) is clinically objectified by the Slocum and Larson test [25]. With the knee in 15° external rotation and 30° flexion, the examiner pulls the tibia forward. The test is positive when the medial joint plate moves forward more than in the contralateral knee. This positive test suggests injury to the ACL, MCL and POL, if the lateral and posterolateral structures of the knee are intact. The Slocum–Larson test must be distinguished from the dial test at 30° flexion, which is positive in the case of posterolateral ligament lesions [9].

The importance of the posterior horn of the MM was emphasised by Werner Muller as early as 1983: ‘The posterior horn of the medial meniscus is an important structure acting as a wheel brake against anterior subluxation of the medial tibial plateau’ [11]. The term ‘ramp lesion’ was introduced by Michael Strobel in 1988 and refers to a lesion of the capsulomeniscal junction at least 2.5 cm in length [27]. In a cadaveric study, Peltier et al. demonstrated a controlling role for ATT, but not for rotation [19]. Ahn et al. demonstrated a significant increase in ATT (2 ± 4.8 mm on average) and IR (2.1 ± 5° on average) after MMPH [1]. Stephen et al. showed a significant increase in ATT (gain of 3 ± 4 mm at 30° flexion and 90 N traction) and ER (gain of 2 ± 1.5° at 30° flexion and 5 N m torque) after a posterior capsulomeniscal lesion but no effect on IR [26]. DePhillipo et al. confirmed gains in ATT (+0.8 mm), IR (+1.3°) and ER (+1°) [7]. In the present study, after the creation of a ramp lesion, there was an increase in translation of 0.9 mm, IR of 0.7° and ER of 0.8°. The MMPH has been considered as a secondary stabiliser for ATT [1, 24, 31].

We acknowledge several limitations inherent in any study in vitro. First, the testing protocol could only quantify the passive contributions to forces and torques, while the muscles remained unloaded. The semi‐membranosus tendon was not studied but it acts as a major active restraint to external rotation with the knee flexed and a protector of the MMPH [15, 21]. Second, the age of the cadaveric knees (average 81 years) was much higher than that of athletes with severe knee sprains. There were two cases at 41 years old, their specific results were not different than with the group of lower limbs around 81 years. The low translation and rotation amplitudes recorded reflect the high age of the donors, but comparisons were also made in VR (%), so we believe that the use of old specimens did not influence the conclusions. Third, all knees were tested only at 30° flexion, imposed by the position on the Dyneelax®. Other anatomical studies tested knees at 0°, 30°, 60° and 90°, such as Ball et al., which is closer to clinical reality, as the role of different structures also varies according to the degree of flexion [2, 9]. Some structures play a crucial role in restraining movement at high flexion angles, but we were unable to confirm this information. Fourth, the sequential cutting protocol (from front to back in all the specimens) used in the present study depends on there being a lack of significant load‐transfer interactions between each of the structures of interest. The cutting order (front to back) could amplify the resisting function of the structure in the back of the medial side with significant load transfer interactions. Fifth, as in all cadaveric studies, the results are only valid at time zero and tissue healing cannot be accounted for. Six, in the transection stage, the sequential cutting order was not randomised, which may be the source of bias. Finally, it should also be noted that we did not test the structures involved in controlling the frontal plane, as the Dyneelax* does not allow valgus/varus stress to be applied.

Conversely, the strengths of the study include the ability to apply repeatable tibial forces and torques (minimum 5 measures) and the precision and accuracy of the device [6]. The loads applied to the knees were similar to those imposed during clinical manual tests, Lachman tests and tests in external and internal rotations, rather than the loads expected in sports activities and dynamic tests in rotations. The sequential cutting protocol used in the present study (ACL then medial structures) could reproduce the translation and rotations of high‐energy knee injuries [2, 14]. For example, the association of ACL and posteromedial corner tear leads to an AMRI. The specimens were prepared without removing the muscles. One of the strengths of our study was the high number of knees tested (29 cases), one of the largest series in the literature. To our knowledge, most of cadaveric studies involved around 10 knees [1, 2, 24, 26]. Our study was always carried out with the knee in 30° flexion, without modifying the knee's position, which allows a slight relaxation of the knee's capsulo‐ligament structures and multidirectional play. We also exhaustively tested all passive ligament structures in the medial side (including the AMR), as well as the MMPH and the ACL.

This present study highlights the specific functions of the ACL and each part of the medial side. Abnormal clinical tests (antero drawer test at 30° and 90° of flexion and external rotation, external rotation dial test at 30° and 90° of flexion) can help to identify which ligaments are injured and which medial procedure (individual reconstruction techniques) should be added to the ACL reconstruction [3]. A positive anterior drawer test in 30° of external rotation indicates a major disruption of the medial ligament and capsular side of the knee (Slocum and Larson test). A combined procedure (ACL plus medial side reconstruction) will protect the ACL graft and avoid a persisting anteromedial rotatory instability. According to the biomechanical literature and the present study, we believe that in cases in which an operative repair or reconstruction is indicated, all injured medial knee structures (including the AMR) should be addressed. Also, this study suggests that an unrepaired ramp lesion will allow abnormal tibiofemoral residual laxity and excessive stress on an ACL reconstruction. It can be recommended that surgeons routinely inspect the posteromedial capsulomeniscal junction to identify and treat these lesions in cases of ACL reconstruction.

## CONCLUSION

The ligamentous structures of the medial plane constitute a functional unit in which each component has a specific passive contribution. The clinical importance of this work is that it shows why isolated ACL reconstruction may not restore knee stability in the case of combined ACL plus severe medial side tears (anteromedial rotatory instability). This study highlights the importance of recognising the extent of the medial injury with specific clinical tests (Lachman test in external rotation) and a meticulous reading of the MRI. It has demonstrated the function of the dMCL plus sMCL plus AMR in restraining tibial external rotation, the contribution of the PMC in resisting internal rotation, and the role of the medial meniscus in controlling translation and rotations. This work has provided data to detect specific lesions in acute or chronic cases and to perform a medial side anatomic reconstruction and a suture of a ramp lesion, in addition to an ACL surgery. Given the different contributions of each of the components of the medial side of the knee, individual reconstruction techniques may be necessary.

## AUTHOR CONTRIBUTIONS

Henri Robert supervised all the steps of the testing protocol. Baptiste Guegan and Henri Robert analysed, interpreted the data and wrote the manuscript. Baptiste Guegan, Michel Drouineau, Harold Common and Henri Robert performed the knee dissection. All authors have read and approved the final version of the manuscript.

## CONFLICT OF INTEREST STATEMENT

The authors declare no conflict of interest.

## ETHICS STATEMENT

Written informed consent from the donors for educational and research purposes was obtained for each specimen.

## Data Availability

The data sets used and analysed during the current study are available from the corresponding author upon reasonable request.
